# Role of Rho-associated kinases and their inhibitor fasudil in neurodegenerative diseases

**DOI:** 10.3389/fnins.2024.1481983

**Published:** 2024-11-19

**Authors:** Qiuyan Ye, Xue Li, Wei Gao, Jiayue Gao, Liping Zheng, Miaomiao Zhang, Fengge Yang, Honglin Li

**Affiliations:** ^1^Graduate School, Heilongjiang University of Chinese Medicine, Harbin, China; ^2^Jiangsu College of Nursing, Huaian, China; ^3^The Second Affiliated Hospital of Heilongjiang University of Chinese Medicine, Harbin, China

**Keywords:** neurodegenerative diseases, Rho-associated kinases, fasudil, Alzheimer’s disease, Parkinson’s disease, Huntington’s disease, amyotrophic lateral sclerosis, multiple sclerosis

## Abstract

Neurodegenerative diseases (NDDs) are prevalent in the elderly. The pathogenesis of NDDs is complex, and currently, there is no cure available. With the increase in aging population, over 20 million people are affected by common NDDs alone (Alzheimer’s disease and Parkinson’s disease). Therefore, NDDs have profound negative impacts on patients, their families, and society, making them a major global health concern. Rho-associated kinases (ROCKs) belong to the serine/threonine protein kinases family, which modulate diverse cellular processes (e.g., apoptosis). ROCKs may elevate the risk of various NDDs (including Huntington’s disease, Parkinson’s disease, and Alzheimer’s disease) by disrupting synaptic plasticity and promoting inflammatory responses. Therefore, ROCK inhibitors have been regarded as ideal therapies for NDDs in recent years. Fasudil, one of the classic ROCK inhibitor, is a potential drug for treating NDDs, as it repairs nerve damage and promotes axonal regeneration. Thus, the current review summarizes the relationship between ROCKs and NDDs and the mechanism by which fasudil inhibits ROCKs to provide new ideas for the treatment of NDDs.

## 1 Search strategy

Several databases including PubMed, Web of Science, and China National Knowledge Network (CNKI) were searched on a computer, and the retrieval time was set to be established until October 2024. Search terms include, but are not limited to, “Neurodegenerative diseases,” “Rho-associated kinase,” and “fasudil,” among others. The inclusion criteria were as follows: Studies that explored the relationship between ROCKs and neurodegenerative diseases and those that demonstrated that fasudil inhibited ROCKs. The exclusion criteria were as follows: Documents that were not relevant to the topic, those were of poor quality, and those that lacked full text access. Finally, 178 articles were included.

## 2 Introduction

Neurodegenerative diseases (NDDs) have become a major public health concern worldwide. Alzheimer’s disease (AD) is the most common NDD, affecting approximately 50 million people, followed by Parkinson’s disease (PD) affecting over 6 million people. Although the prevalence of Huntington’s disease (HD), amyotrophic lateral sclerosis (ALS), and multiple sclerosis (MS) is relatively low, they still warrant further exploration. Currently, the number of patients with AD and PD alone has exceeded 20 million worldwide. Age is the most important factor in NDDs. The annual prevalence of NDDs is rising steadily with a rapidly aging population, seriously affecting people’s daily lives and social development (Koch et al., 2018). Consequently, preventing and delaying the progression of NDDs is paramount.

Ras homolog guanosine triphosphatases (Rho GTPases), which are part of the Ras superfamily, play a crucial role in diverse cellular processes such as cell morphology, polarity, adhesion motility, and membrane protrusion, acting as a molecular switch by cycling between the active state of guanosine triphosphatases (GTP) binding and the inactive state of guanosine diphosphate (GDP) binding (Beljan et al., 2020; Reiner and Lundquist, 2018). Activated Rho GTPases regulate cellular processes by binding to the downstream effector proteins and participating in downstream signaling cascades (Vega and Ridley, 2008). Rho-associated kinases (ROCKs) are downstream targets of Rho GTPases (Matsui et al., 1996). The combination of Rho GTPases and GTP can activate ROCKs, enabling them to play a crucial role in cell proliferation, adhesion, contraction, secretion, and apoptosis (Wei et al., 2021). The ROCK signaling is a major driver of numerous human diseases and its activation enhances the occurrence of NDDs (Henderson et al., 2016; Ohashi et al., 2000; Shao et al., 2008a,b). Therefore, inhibition of ROCKs is the current research hotspot. Fasudil is an inhibitor of ROCKs with high safety and it was first approved in Japan for the treatment of vasospasm after subarachnoid hemorrhage (Roskoski Jr, 2023). In recent years, numerous studies have demonstrated that fasudil has a protective effect against multiple NDDs and is considered a potential drug for treating NDDs. Hence, the present review explores the relationship between ROCKs and NDDs and the mechanism through which fasudil inhibits ROCKs to improve neurodegeneration.

## 3 Structure and overview of ROCKs

Rho GTPases, which are part of the Ras superfamily, including RhoA, RhoB, and RhoC isoforms, are essential in the structural domains of mammals and eukaryotes and participate in regulating actin cytoskeletal remodeling such as cell morphology, polarity, adhesion motility, and membrane protrusion (Beljan et al., 2020; Fort and Blangy, 2017). Most Rho GTPases are regulated by guanine nucleotide dissociation inhibitors (GNDIs), Rho GTPase-activating proteins (GAPs), and guanine nucleotide exchange factors (GEFs). These proteins control the switching of GDP-bound inactive and GTP-bound active states, functioning as molecular switches (Cord, 2021; Roskoski Jr, 2023; Reiner and Lundquist, 2018). Activated Rho GTPases regulate cellular processes by binding to and regulating downstream effector proteins such as ROCKs (Vega and Ridley, 2008).

**Figure 1:**
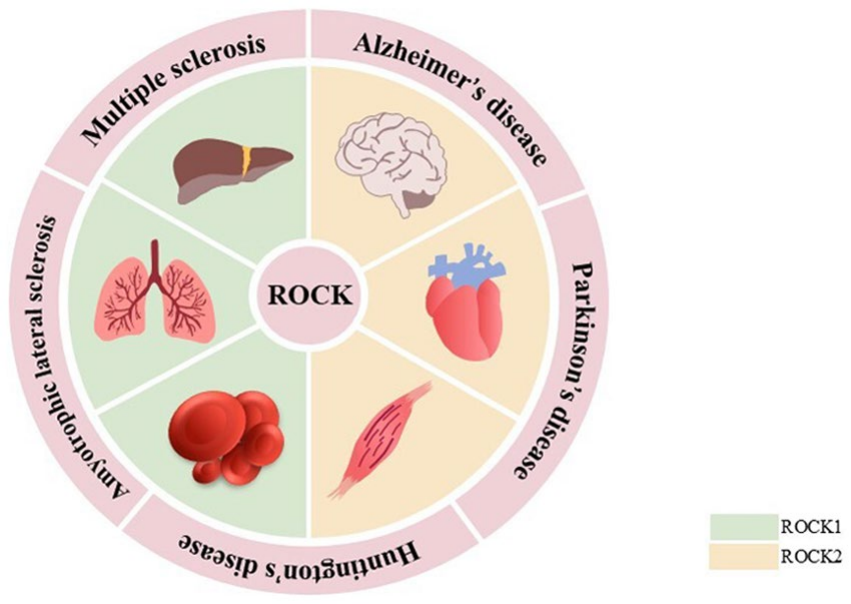
Expression of ROCKs and the relationship between ROCKs and neurodegenerative diseases.

**Figure 2:**
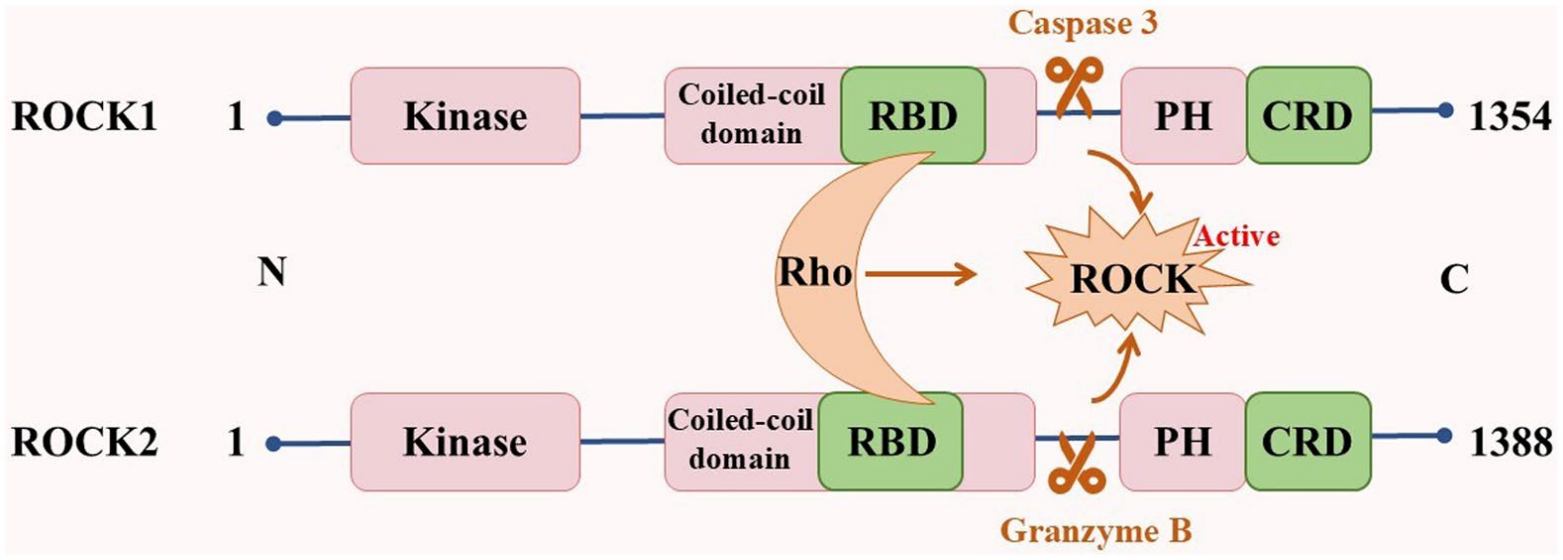
Structure of ROCKs.

ROCKs, which are members of the cAMP-dependent protein kinase A, cGMP-dependent protein kinase G, and phospholipid-dependent protein kinase C (AGC) protein kinase family, are serine/threonine protein kinases with a molecular weight of ~160 kDa. They are composed of an N-terminal kinase domain, a Rho-binding domain (RBD), a central coiled-coil domain, a Pleckstrin-homology domain (PH), and a cysteine-rich domain (CRD; Amano et al., 2010). There are two subtypes of ROCKs: ROCK1 and ROCK2 (Shahbazi et al., 2020). Although both ROCK1 and ROCK2 are universally expressed, ROCK1 is mainly expressed in non-neuronal tissues such as the liver, lung, and blood, whereas ROCK2 is mainly expressed in the brain, heart, and muscle (Julian and Olson, 2014; Mani et al., 2022; Figure 1). The kinase domain homology of these two subtypes is 92%, the coiled-coil domain homology is 55%, and the entire amino acid sequence similarity is 65% (Nakagawa et al., 1996). These properties suggest that their functions are highly similar. The serine/threonine LIM domain kinase (LIMK), myosin light chain phosphatase (MLCP), myosin phosphatase-targeting subunit 1 (MYPT1), and other downstream targets regulate cellular processes such as actin cytoskeleton, stress fibers, and cell contraction. However, ROCK1 and ROCK2 features are non-redundant and unique. In cellular processes, ROCK1 is essential for the formation of stress fibers and adhesion plaques, whereas ROCK2 is essential for phagocytosis and cell contraction (Wang et al., 2009; Yoneda et al., 2005). ROCK1 knockdown reduces cell migration and proliferation and reshapes cell morphology. However, ROCK2 knockdown changes the cell migration ability (Mertsch and Thanos, 2014). In subcellular localization, ROCK1 is mainly localized to actin filaments, whereas ROCK2 is localized to membrane processes. Both kinases differentially regulate dendritic spine morphology by regulating different pathways in cell polarity (Newell-Litwa et al., 2015; Yan et al., 2019). In addition, ROCKs possess a self-inhibiting zone in most cases, allowing ROCKs to exist in the cytoplasm in an inactive form in a self-inhibiting state. When Rho binds to RBD or C-terminal cleavage (caspase 3 cleavage activates ROCK1, whereas granzyme B cleavage activates ROCK2), it induces the activation of ROCKs, which leads to various diseases (Cai et al., 2021; Craft Jr et al., 2013; Hahmann and Schroeter, 2010; Figure 2).

## 4 Role of ROCKs in NDDs

### 4.1 Alzheimer’s disease

AD, an age-related progressive NDD, is the most prevalent form of dementia, accounting for approximately 70% of all dementia cases (Mani et al., 2022). According to recent statistics, approximately 50 million people are affected by AD globally, which may increase to 131 million by 2050 (Whitaker et al., 2014). The initial symptom of AD is typically a decline in recent memory function, accompanied by clinical manifestations such as attention deficits, spatial disorders, and behavioral impairments (Cai et al., 2021). The pathogenesis of AD remains unclear, and the mainstream hypotheses include amyloid-*β* (Aβ), tau pathology, and synaptic dysfunction. AD is often manifested by the formation of extracellular senile plaques caused by Aβ deposition, intracellular neuronal fibrillary tangles (NFTs) caused by over-phosphorylation of tau protein (p-tau), and neuronal loss (Jack et al., 2018; Zhang et al., 2023).

Amyloid plaque deposition is the most significant pathological feature of AD (Lim et al., 2012). Studies have shown that Aβ promotes ROCK1 activity in neurons; abnormal activation of ROCKs has also been observed in the brains of AD patients and AD mice (Gao et al., 2019; Henderson et al., 2016; Hu et al., 2019). This may be caused by the interaction between Aβ, RhoA, and N-methyl-D-aspartic acid receptors to activate ROCKs (Lacor et al., 2007; Petratos et al., 2008; Pozueta et al., 2013). Aβ activates the RhoA/ROCK pathway in AD brains, significantly elevating ROCK1, which in turn promotes the rate of phosphorylation of the amyloid precursor protein (APP) phosphorylation by beta-site amyloid precursor protein cleaving enzyme 1 (BACE1), accelerating Aβ production (Henderson et al., 2016; Hu et al., 2019). ROCK1 knockdown reduces Aβ levels in neurons (Henderson et al., 2016). Previous studies have found that ROCK2 inhibition decreased BACE1 activity and suppressed Aβ40 production in the 5 × FAD mouse model (Herskowitz et al., 2013). In addition, Nogo-A is a myelin-associated protein that can inhibit axon regeneration and neurite growth (Chen et al., 2000; Pernet and Schwab, 2012; Sekine et al., 2020). Activation or overexpression of Nogo-A and its receptor NgR can activate ROCKs to inhibit neurite growth and promote Aβ production (Xiao et al., 2012). Inhibiting ROCKs and blocking their associated pathways reduces Aβ deposition and is beneficial to nerve and myelin regeneration (Guo et al., 2020a; Li et al., 2017). Collectively, these pieces of evidence suggest that Aβ and ROCK interaction can lead to a vicious cycle, exacerbating nerve injury. The inhibition of ROCKs may be an effective way to reduce Aβ deposition and neurotoxicity.

The C-terminal of tau can inhibit tau aggregation *in vitro*. The cysteine aspartate protease caspase can cleave the C-terminal of tau, increasing tau aggregation and cytotoxicity (Berry et al., 2003; Gamblin et al., 2003). It has also been previously reported that tau is phosphorylated by ROCKs at Thr245, Thr377, and Ser409 (Amano et al., 2003). The inhibition of ROCKs was found to promote caspase-3 inactivation while activating tau autophagy and degradation, reducing tau aggregation, p-tau, and NFTs, and improving learning and spatial memory (Castro-Alvarez et al., 2011; Gentry et al., 2016; Hamano et al., 2020). Protein kinase B (AKT) phosphorylates glycogen synthesis kinase 3β (GSK-3β) to inactivate it and play a role in protecting neurons (Chu et al., 2017; Wu et al., 2012). The inhibition of ROCKs indirectly activates AKT by down-regulating tau kinases GSK3*β* and cyclin-dependent kinase 5 (Cdk5), thereby reducing p-tau protein levels (Hamano et al., 2020). ROCK1 or ROCK2 knockdown in cells also significantly reduces tau protein and messenger RNA expression levels (Gentry et al., 2016). Therefore, ROCKs are involved in tau pathological changes and inhibition of ROCKs can improve tau protein disease.

Synaptic function is closely related to memory ability and its dysfunction is an important cause of cognitive decline in AD. Actin and dendritic spines are tightly linked to cytoskeletal remodeling and normal synaptic function. It is well-established that Aβ activates ROCKs. After activation, ROCK1 negatively regulates dendritic spine length through the myosin-actin pathway. ROCK2 inhibits dendritic spine density through the serine/threonine LIM domain kinase isoform 1 (LIMK1)-phosphorylated cofilin1 (p-cofilin) signaling pathway (Martín-Cámara et al., 2021; Yan et al., 2019). Meanwhile, abnormal expression of cofilin1 also leads to the formation of a cofilin-actin rod, which is conducive to the formation of NFTs by tau protein and further aggravates neuronal apoptosis (Bamburg et al., 2010; Cichon et al., 2012). Tau is one of the factors that cause synaptic dysfunction (Lasagna-Reeves et al., 2011; Meraz-Ríos et al., 2010). The inhibition of ROCKs can effectively reduce p-tau and oligomeric tau (Hamano et al., 2020). The classical Wnt signaling pathway (Wnt-β/catenin) facilitates synaptic formation and stabilization, whereas the non-classical Wnt signaling pathway (Wnt-PCP) promotes synaptic depolymerization (Nagaoka et al., 2014; Stamatakou and Salinas, 2014). Aβ can activate the Wnt-PCP/RhoA/ROCK pathway by the upregulating the Dickkopf-1 expression, leading to retraction of the dendritic spine and disruption of synaptic homeostasis (Sellers et al., 2018). Additionally, ROCKs can activate myosin-II by phosphorylating the MLCP and MYPT1 formation of actin arcs to inhibit microtubule protrusion in growth cones (Dupraz et al., 2019). Meanwhile, ROCK2 can collapse the growth cone by phosphorylating collapsin response mediator protein 2 (CRMP2). It can also induce growth cone collapse by phosphorylating CRMP2, which explains the causative mechanisms of ROCKs on other NDDs (Salminen et al., 2008). Studies have shown that ROCKs can regulate neuronal polarization and axon growth through the cytoskeleton, leading to neurodegeneration (Henderson et al., 2019; Martín-Cámara et al., 2021; Zhang H. et al., 2021).

Neuroinflammation plays an important role in neurodegeneration and cognitive dysfunction. ROCKs are potential targets for treating neuroinflammation and play a role in regulating neuroinflammation (Shinozaki et al., 2019). Microglia play an important role in the stability of the central nervous system. There are two types of microglia; M1, which expresses oxidative stress and inflammatory factors, and M2, which contains anti-inflammatory and tissue repair factors (Zhang et al., 2013). Physiologically, microglia can phagocytose Aβ (Wang et al., 2021). Activation of the RhoA/ROCK pathway inhibits microglia from the phagocytosis of Aβ and leads to microglia dysfunction, triggering an inflammatory response, leading to a vicious cycle of neuroinflammation and neurotoxicity (Wang et al., 2021; Zhang et al., 2019). Nuclear factor kappa B (NF-κB) is a crucial transcription factor that modulates inflammatory response, and GSK3β forms a bridge between Aβ and tau (Hooper et al., 2008; Tornatore et al., 2012). Besides targeting microglia, Aβ activates the RhoA/ROCK pathway to promote inflammation, Aβ production, and tau phosphorylation (Ewers et al., 2019; Jiang et al., 2015; Morenas-Rodríguez et al., 2022; Xie et al., 2015; Zhang et al., 2023). Therefore, inhibition of ROCKs can alleviate neuroinflammation and nerve damage (Tseng et al., 2019; Zhu et al., 2020).

In summary, these findings collectively suggest that ROCKs play a multifaceted role in the progression of AD, mediating the disease through various pathways and interacting pathological markers to exacerbate AD injury. Targeting ROCKs may represent an effective strategy to delay AD progression and alleviate its symptoms.

### 4.2 Parkinson’s disease

PD, the second most common NDD after AD, is a degenerative movement disorder caused by progressive degeneration of dopamineric (DA) neurons in the dense bodies of the substantia nigra (SN) of the midbrain (Ye et al., 2023). Bradykinesia, resting tremor, and muscle rigidity are the main motor features of PD (Schmidt et al., 2022). Currently, there are over 6 million cases of PD worldwide and the pandemic is growing exponentially (Dorsey et al., 2018; Morens et al., 2009). The prevalence of PD is increasing with an increase in the global aging population, with a projected increase to over 12 million by 2040 (Dorsey and Bloem, 2018).

Axonal degeneration is one of the earliest features of PD, appearing in the early stages of PD (Iyer et al., 2021). The RhoA/ROCK signal transduction can stimulate LIMK1, inactivate cofilin, lead to actin waves, axon elongation, and growth cone disorders, and mediate the axonal degeneration of PD and DA loss (Ohashi et al., 2000; Tilve et al., 2015; Takemura et al., 2009; Wang and Townes-Anderson, 2016). The toxic damage of neurons caused by *α*-synuclein (α-Syn) accumulation is one of the main causes of PD. A previous study reported ROCK2 activation and neurite reduction in A53T α-Syn-induced neurite growth injury, which was alleviated by the inhibition of ROCKs (Liu et al., 2016). This indicates an interaction between ROCKs and *α*-Syn, which can damage neurites and accelerate the PD process.

The degeneration of DA neurons in the nigrostriatal pathway is characteristic of PD and can lead to movement disorders typical of PD (Moore et al., 2005). Clinical studies have shown that about 60% of nigrostriatal dense neurons are lost and about 80% of DA endings are dysfunctional in PD patients (Bernheimer et al., 1973). 1-Methyl-4-phenyl-1,2,3,6-tetrahydropyridine (MPTP) can reduce the expression of dopamine transporter and induce the degeneration of DA neurons (Muroyama et al., 2011). Previous studies found that the activity of ROCKs increased and DA was lost after MPTP injection. Moreover, the knockdown or inhibition of ROCKs effectively alleviated MPTP-induced neuronal degeneration and protected DA neurons (Barcia et al., 2012; Qi et al., 2016). This indicates that ROCKs induce DA neuronal degeneration (Zhao et al., 2015). In addition, MPTP-induced neuronal mitochondrial autophagy in PD mice was insufficient and the damaged mitochondria accumulated excessively in the substantia nigra and striatum (Zhang L. et al., 2021). Parkin (an E3 ubiquitin ligase) is a key factor regulating mitochondrial autophagy, and ROCKs are negative regulators of Parkin-dependent mitochondrial autophagy (Mani et al., 2022). Deletion of Parkin can lead to further aggregation of *α*-Syn, damage mitochondrial function, and exacerbate synaptic dysfunction and neuronal damage (Chen L. et al., 2015; Volpicelli-Daley, 2019; Xu et al., 2020). The inhibition of ROCKs increases the activity of hexokinase 2 (HK2), a positive regulator of Parkin, relocating it to the mitochondria, thereby promoting mitochondrial autophagy (Moskal et al., 2020; Quadir et al., 2021). This suggests that mitochondrial autophagy can eliminate defective mitochondria, and the accumulation of damaged mitochondria may aggravate the death of DA neurons. In conclusion, ROCKs may aggravate DA loss by accelerating neuronal degeneration and mitochondria autophagy. Therefore, targeted inhibition of ROCKs may be one of the potential strategies to reduce PD damage.

ROCK activity is associated with neuroinflammation. Previous studies observed activated microglia and reactive astrocytes in the brains of PD patients, and the expression of ROCK2 in these cells was increased (Kam et al., 2020; Zaman et al., 2021). ROCKs are important for α-Syn clearance and metabolism, and treatment with ROCK inhibitors reduces the accumulation of α-Syn (Liu et al., 2016; Martín-Cámara et al., 2021). These results suggest that α-Syn accumulation is positively correlated with enhanced ROCK activity. In addition, α-Syn can bind to the integrin cluster of differentiation molecule 11b (CD11b) to activate the downstream Rho/ROCK signaling pathway, which induces nicotinamide adenine dinucleotide phosphate oxidase 2 (NOX2) activation and leads to reactive oxygen species production (Cap et al., 2020; Vermot et al., 2021). Excessive production of reactive oxygen species can lead to neuronal damage and oxidative stress in PD patients (Chang and Chen, 2020; Xiao et al., 2022). Furthermore, oxidative stress can induce the release of proinflammatory factors, thus exacerbating neuronal damage and apoptosis (Gathings et al., 2024). The specific toxicity of DA neuronal damage subsequently induces microglia polarization, resulting in the phagocytosis of DA cell bodies. The inhibition of ROCKs can prevent microglia polarization, reduce the release of inflammatory factors, and suppress the loss of DA neurons (Barcia et al., 2012; Quadir et al., 2021).

Therefore, ROCKs may be both a pathologic product of PD and a promoter of PD progression. Early intervention of the expression of ROCKs may be a feasible approach to prevent and treat PD.

### 4.3 Huntington’s disease

HD is a dominant NDD with the main clinical manifestations including dance-like movement, mental decline, and mental behavior abnormalities (McColgan and Tabrizi, 2018). HD is caused by amplified CGA codon repeats of elongated polyglutamine (polyQ) in the huntingtin protein (Htt). An amplified polyQ is toxic, and there is currently no effective treatment for it (Shao et al., 2008a). Patients cannot take care of themselves after HD onset, imposing a substantial burden on the family and society.

The actin-binding protein profilin is an Htt-interacting protein that inhibits Htt aggregation in mutants. The Rho/ROCK signaling phosphorylates ROCKs in nerve and non-nerve cells at Ser137, thereby blocking profilin phosphorylation and reducing Htt-mediated toxicity (Li et al., 2013; Shao et al., 2008a). It has also been shown in HD cells and drosophila models that elevated ROCK expression enhances polyQ aggregation, whereas ROCKs inhibition reduces polyQ aggregation and its toxicity (Bauer et al., 2009; Pollitt et al., 2003; Shao et al., 2008b). Compared with the control group, HD patients exhibited increased ROCK1 expression levels in the frontal cerebral cortex and blood. The level of ROCK1 protein was also significantly increased in R6/2 HD model mice (Narayanan et al., 2016). This suggests that ROCK1 plays a crucial role in HD lesions. In addition, the striatum is the primary site of HD degeneration. D2 receptor stimulation and formation of aggregation can indirectly activate the RhoA/ROCK pathway and disrupt neurite and growth cone formation, thereby exacerbating striatal damage (Deyts et al., 2009). Antagonistic D2 can inhibit the striatal protection function of RhoA/ROCK (Charvin et al., 2005). Therefore, ROCKs are an important factor that promote the progression of HD, and its inhibiting may delay the progression of HD. However, reports on the relationship between ROCKs and HD are limited, with available studies mainly focusing on ROCK1, and the specific mechanism remains elusive.

### 4.4 Amyotrophic lateral sclerosis

ALS is a rare NDD that affects middle-aged and older adults, with a global age-standardized incidence of approximately 1.68 per 100,000 people per year (Marin et al., 2018). ALS is characterized by degeneration of upper and lower motor neurons at the spinal cord or medulla oblongata level, resulting in muscle weakness and atrophy, dysarthria, dysphagia, and death from respiratory failure (Hardiman et al., 2017).

Mutations in superoxide dismutase 1 (SOD1) have been associated with ALS. Previous studies found that the protein levels of ROCK2 and its downstream targets LIMK1 and cofilin2 were significantly increased in the skeletal muscle of ALS patients compared with the control group of the same age (Conti et al., 2014). Similarly, RhoA and ROCK2 were abnormally expressed in SOD1 mutant mice (Liang et al., 2020). These results suggest that the Rho/ROCK signaling pathway may be involved in ALS and motor neuron degeneration. In addition, phosphorylated AKT levels were reduced in motor neuron cells of ALS patients and SOD1 mutant mice in the early stages of the disease (Dewil et al., 2007). The mechanism may be that ROCKs inhibit AKT phosphorylation by phosphorylating phosphatase and tensin homolog (PTEN), which is involved in SOD1-induced motor neuron cell death. However, ROCK inhibitor treatment reduces neuronal death and alleviates ALS axon regeneration and motor injury, which delays the disease process (Günther et al., 2017; Joshi et al., 2019; Takata et al., 2013; Tönges et al., 2014). Thus, ROCKs induce motor neuron cell death in ALS (Stankiewicz et al., 2020).

ROCKs are an important factor in ALS, which may mediate the degeneration of motor neurons through the PTEN/AKT pathway or actin. The inhibition of ROCKs may reduce motor neuron injury and delay the process of ALS.

### 4.5 Multiple sclerosis

MS is a chronic autoimmune inflammatory disease of the central nervous system, characterized by demyelination, axon damage, and neurodegeneration (Garg and Smith, 2015). Currently, about 2.8 million people worldwide live with MS. MS etiology remains unknown and there is currently no cure for this disease (The Lancet Neurology, 2021). Axon damage is the main cause of irreversible neurological disability in MS. A previous study found that ROCK activity increased in the serum of MS patients and mice compared with the control group. Moreover, serum co-cultures revealed a shortening of neurites and decreased cell activity. However, treatment with ROCK inhibitors promoted neuron growth and synaptic formation (Chen C. et al., 2015). This suggests that MS axon loss is associated with increased ROCK activity, and inhibition of ROCKs is an effective strategy to prevent synaptic damage and promote nerve recovery. In addition, myelin degradation can lead to loss of axon function and eventually translate to axonal degeneration. Differentiation of resident oligodendrocyte precursor cells (OPCs) can regenerate the exfoliated axonal myelin sheath in early stages of MS. Therefore, enhancing endogenous OPC maturation and myelin regeneration is an effective therapeutic strategy for MS. Intriguingly, the Rho/ROCK signaling pathway can directly or indirectly participate in oligodendrocyte maturation and myelination, and the inhibition of ROCKs can promote the differentiation and myelination of OPCs (Paintlia et al., 2008; Pedraza et al., 2014). Therefore, the inhibition of ROCKs may improve MS damage by repairing synapses and promoting myelin regeneration.

**Figure 3:**
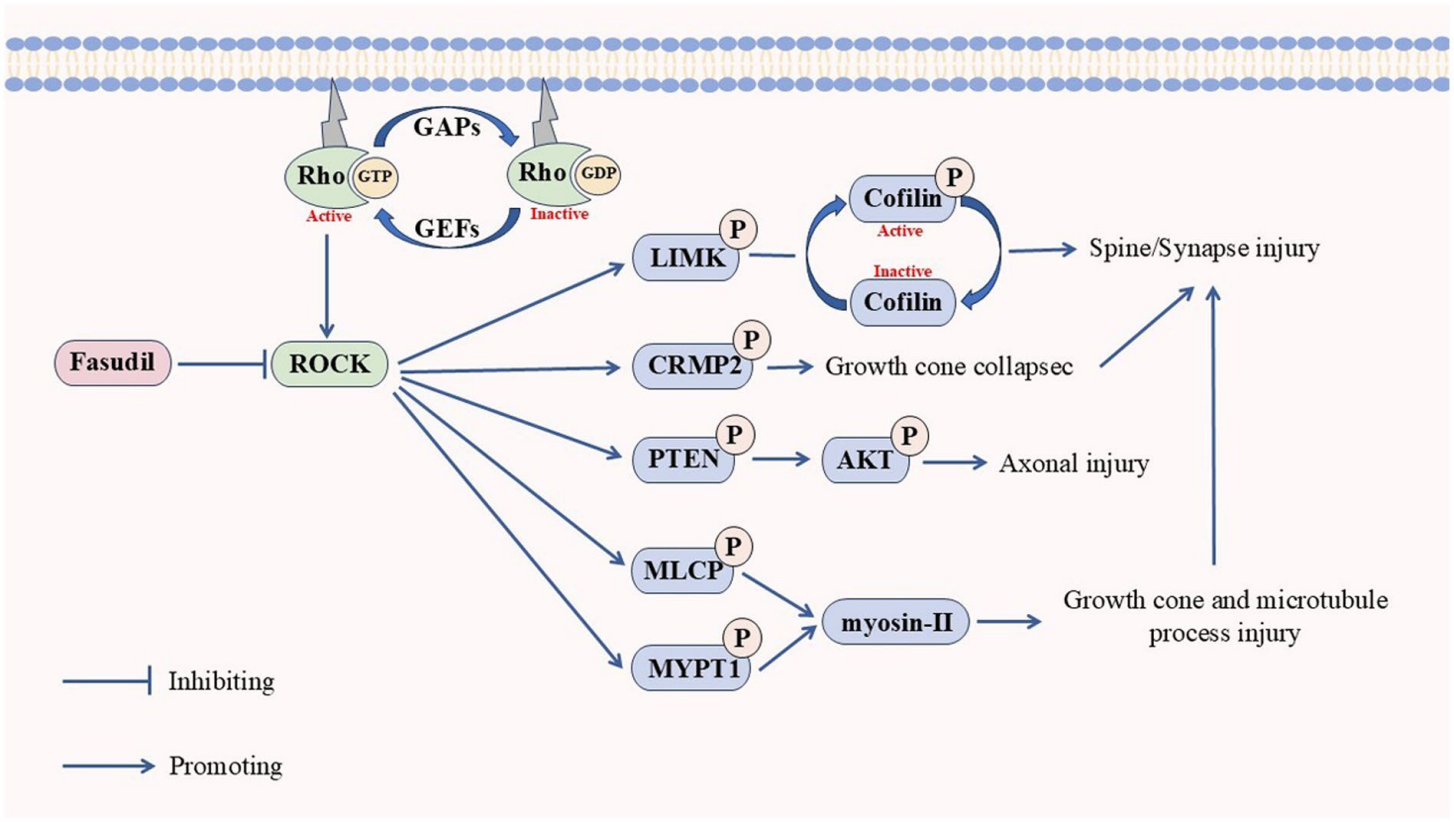
Pathways associated with neurodegenerative diseases caused by ROCKs.

Overall, ROCKs are shared signaling kinases in multiple NDDs, and elevated ROCK activity may be a potential biomarker for NDDs. Inhibition of ROCKs promotes nerve regeneration and can improve NDDs symptoms regarding nerve damage and synapses (Figure 3).

## 5 Application of ROCKs inhibitor fasudil in NDDs

ROCKs are potential targets for treating NDDs, and ROCK inhibitors have neuroprotective effects. In recent years, over 200 ROCK inhibitors have been identified in the clinical trial stage, of which 75 have been approved by the US Food and Drug Administration (FDA) and 21 have been approved in China, Japan, and South Korea. Among them, fasudil acts by competing for adenosine triphosphate (ATP)-binding sites in the ROCKs catalytic domain, and is the first approved ROCKs protein kinase inhibitor, officially approved in Japan in 1995 for the treatment of vasospasm after subarachnoid hemorrhage (Roskoski Jr, 2023). Clinical trials have shown that fasudil is well tolerated, with few side effects and no major safety concerns (Vicari et al., 2005). However, fasudil has a short time window and cannot be taken for a long time. Therefore, efforts have been made to identify new ROCK inhibitors, including derivatives of fasudil. For instance, hydroxyfasudil and dimethylfasudil can protect nerves and promote nerve regeneration and functional recovery (Lingor et al., 2007; Rikitake et al., 2005; Sezen et al., 2014). Y-27632 is also a typical ROCK inhibitor that is often used in cell and animal model studies (Kuroda et al., 2015; Miyagi et al., 2019; Pinca et al., 2017; Rodríguez-Trillo et al., 2022; Wang et al., 2017). Lingor et al. (2007) compared the effectiveness of fasudil, dimethylfasudil, and Y-27632 in inhibiting ROCK-promoted neuronal growth. The study found that all three inhibitors promoted neuron growth; however, the effects of fasudil and Y-27632 increased in a concentration-dependent manner. Overall, dimethylfasudil has the smallest concentration range, the best inhibition effect, and the highest level of safety. However, the treatment window of dimethylfasudil is narrow and the curative effect is limited owing to the permeability, chemical stability, and biodegradation of the membrane. The most common ROCKs inhibitors at present are summarized in Table 1. Ki results show that fasudil has a strong inhibitory effect. Therefore, fasudil remains one of the most competitive ROCK inhibitors currently approved.

**Table 1:** Common ROCKs inhibitors.

Inhibitor	Target	Indication	Ki(μM)	References
Fasudil	ROCKs PKA PKC PKG MLCK	Vasospasm after subarachnoid hemorrhage Huntington’s disease Bladder cancer Epilepsy X-linked intellectual disability Cognitive impairment Amyotrophic Lateral Sclerosis Cardiovascular disease Hepatic fibrosis	ROCK1-0.33 ROCK2-0.158 PKA-4.58 PKC-12.30 PKG-1.650 MLCK-36	Abe et al. (2014); Busti et al. (2020); Deng et al. (2016); He et al. (2017); Koch et al. (2020); Li et al. (2013); Ming et al. (2010); Zhao et al. (2006); Takata et al. (2013); Xi and Xu (2021)
Hydroxylfasudil	ROCKs	Diabetic erectile dysfunction Ischemic stroke Hypertension Vasospasm after subarachnoid hemorrhage Allergic asthma	ROCK1-0.73 ROCK2-0.72	Inoue et al. (2012); Sezen et al. (2014); Rikitake et al. (2005); Wang and Jiang (2020); Satoh et al. (2012); Franova et al. (2023)
Dimethylfasudil	ROCKs	Promote neurite growth	ROCK1-0.0413 ROCK2-0.008	Lingor et al. (2007); Lie et al. (2010)
Netarsudil	ROCKs	Glaucoma	ROCK1-0.0037 ROCK2-0.0023	Ha et al. (2022)
Ripasudil	ROCKs	Glaucoma Ocular hypertension	ROCK1-0.051 ROCK2-0.019	Kaneko et al. (2016); Araie et al. (2023)
Sovesudil	ROCKs	Glaucoma	ROCK1-0.0037 ROCK2-0.0023	Ha et al. (2022)
Y-27632	ROCKsPKN PKCα PKA	Rheumatoid arthritis Fatty liver ischemia/reperfusion injury Corneal endothelial injury Juve sarcoma Systemic lupus erythematosus	ROCK1-0.22 ROCK2-0.3 PKN-3.1 PKCα-73 PKA-25	Rodríguez-Trillo et al. (2022); Kuroda et al. (2015); Miyagi et al. (2019); Pinca et al. (2017); Wang et al. (2017)
Belumosudil	ROCK2	chronic graft-versus-host disease systemic sclerosis	ROCK1-24 ROCK2-0.105	Blair (2021)
GSK429286 A	ROCK1	Allergic asthma Promote angiogenesis Restore limb ischemic blood flow	0.01414	Gondáš et al. (2024); Fayed et al. (2023)
RKI-1447	ROCKs	Breast cancer Rectal cancer Nonalcoholic fatty liver disease	ROCK1-0.0145 ROCK2-0.0062	Patel et al. (2012); Li et al. (2020); Wang and Jiang (2020)

Ki indicates the potency of the inhibitor, which a lower Ki, indicating a stronger inhibitory effect.

It has been demonstrated that ROCKs contribute to synaptic function and inflammatory response (Lee et al., 2022; Shapiro et al., 2019). In addition, the factors that drive the development of NDDs are also implicated in synaptic loss and neuroinflammation (Guzman-Martinez et al., 2019). Animal models have demonstrated that fasudil has beneficial effects on various NDDs. Moreover, a positive correlation was reported between axon injury and inflammatory microglia/macrophage activation (Herz et al., 2010; Lou et al., 2018). Several studies have reported that fasudil suppresses the secretion of proinflammatory factors by converting microglia/macrophages from M1 to M2, which inhibits inflammatory signaling cascades (Barcia et al., 2012; Liu et al., 2024; Zhao et al., 2015). Synaptic plasticity forms the regulation of learning and memory. Fasudil was reported to promote axon and myelin regeneration by regulating actin cytoskeleton and other factors that cause axon disarrangement and synaptic destruction (Jing et al., 2024; Zhu et al., 2022). The effectiveness of fasudil in the treatment of various NDDs is summarized in Table 2.

**Table 2:** Mechanisms of fasudil in the treatment of neurodegenerative diseases.

Disease	Mechanism	References
AD	Fasudil reduces apoptosis and synaptic loss through ASK/JNK, Dkk1/Wnt and other signaling pathways, reduces Aβ load, p-tau and NFTs levels.	Gao et al. (2019); Sellers et al. (2018); Elliott et al. (2018)
	Fasudil inhibited the RhoA/ROCK pathway by regulating Nogo-A/NgR complex and clu protein, reduced the level of Aβ plaques and tau protein, maintained synaptic function and inhibited neuronal apoptosis.	Guo et al. (2020a); Hamano et al. (2020); Giunti et al. (2023)
	Fasudil treats AD by reshaping gut microbial metabolism	Yan et al. (2021)
	Fasudil reduces hippocampal neuronal degeneration and improves learning and memory deficits by inhibiting inflammatory responses.	Song et al. (2013); Guo et al. (2020b)
PD	Fasudil activates macroautophagy through JNK1/Bcl2/beclin1 pathway, reduces α-Syn content and attenuates neurite growth injury caused by A53T α-Syn overexpression.	Tatenhorst et al. (2016); Liu et al. (2016)
	Fasudil protects dopaminergic neurons and promotes their regeneration by inhibiting ROCK/GSK-3β activity, improving PD motor function, and inhibiting inflammatory response and oxidative stress.	Barcia et al. (2012); Zhao et al. (2015); Tönges et al. (2012); Tatenhorst et al. (2014)
	Fasudil alleviates PD injury by inhibiting levodopa-induced dyskinesia.	Lopez-Lopez et al. (2020)
HD	Fasudil alleviates profilin phosphorylation by blocking Htt binding to ROCKs and improves retinal function in HD mice.	Shao et al. (2008a)
	Fasudil inhibits SOD1^G93A^ induced motor neuron cell death by inhibiting ROCKs regulated PTEN/AKT signaling pathway.	Takata et al. (2013)
	Fasudil mitigated mitochondrial dysfunction and neurotoxicity and inhibited degeneration in 3-NP HD rats.	Ahmed et al. (2016)
ALS	Oral administration of fasudil improved motor behavior in male PD mice.	Günther et al. (2017)
	Fasudil inhibits the release of proinflammatory cytokines and chemokines by regulating the activation of RhoA/ROCK and microglia cells, prolongs the survival cycle of ALS and improves motor function.	Tönges et al. (2014)
MS	Fasudil inhibits inflammatory cell infiltration and improves demyelination.	Liu et al. (2024)
	Fasudil promotes microglia to clear pathological myelin debris by up-regulating microglia TREM2/DAP12 pathway, induces neurotrophic factor expression, and promotes myelin regeneration.	Ding et al. (2021)

AD, Alzheimer’s disease; PD, Parkinson’s disease; HD, Huntington’s disease; ALS, Amyotrophic Lateral Sclerosis; MS, Multiple sclerosis; Aβ, beta-amyloid; P-tau, Phosphorylated tau.

### 5.1 Alzheimer’s disease

The results of the Morris water maze test indicated that the platform latency and residence time of the target region of APP/PS1 mice increased following fasudil treatment, implying that fasudil alleviated the learning and memory disorders in AD mice (Yan et al., 2021). Other scholars have indicated that fasudil can suppress endogenous Aβ production and decrease the levels of soluble Aβ and age-plaque deposits in the brains of AD mice, thereby improving Aβ-induced spatial learning and memory impairment (Killick et al., 2023; Song et al., 2013; Yan et al., 2021). The administration of fasudil significantly reduced the Aβ load and tau phosphorylation in primary AD neurons, accompanied by increased expression of anti-apoptotic factor Bcl-2, thereby protecting neuronal processes (Gao et al., 2019). This implies that fasudil inhibits dendritic spines and synaptic barriers and promotes the formation of dendritic branches of neurons (Elliott et al., 2018; Guo et al., 2020b; Hooper et al., 2008). Endothelial cells have been shown to maintain the integrity of the blood–brain barrier (BBB). Oligo-Aβ and oligo-tau can disrupt the integrity of the BBB by activating the RhoA/ROCK signaling pathway via its effects on brain endothelial cells, causing neuroinflammation and degenerative changes (Hossen et al., 2024a). Fasudil treatment was observed to enhance the integrity and permeability of the BBB in AD by inhibiting this signaling pathway. It also decreases oxidative stress, proteasome activity, and mitochondrial dysfunction (Collu et al., 2024; Hossen et al., 2024b). In addition, transcriptomic analysis showed that fasudil suppressed tau phosphorylation by regulating the expression level of cluster proteins and upregulating the expression of AKT serine/threonine protein kinase 1 (Giunti et al., 2023). Neuroinflammation has been found to be strongly associated with AD development. Fasudil prevents the production of IL-1β and TNF-*α* and the activation of NF-κB in AD, which alleviates brain inflammatory damage (Song et al., 2013). Therefore, inhibition of ROCKs by fasudil appears to be an effective strategy for blocking the progression of AD.

### 5.2 Parkinson’s disease

α-Syn is an important pathological marker of PD. Fasudil treatment inhibited α-Syn aggregation by directly binding to the C-terminal of α-Syn, and long-term fasudil-dependent treatment significantly improved the motor and cognitive impairment of α-Syn^A53T^ mice (Tatenhorst et al., 2016). It also activated macrophage autophagy via the JNK1/Bcl2/beclin1 pathway, downregulated the expression of α-Syn levels, and alleviated neuronal growth induced by α-Syn overexpression in A53T (Liu et al., 2016). Thus, fasudil can inhibit α-Syn through various mechanisms and improve PD symptoms. In MPP and MPTP-induced PD models, fasudil improved the motor function of MPTP mice by increasing the survival rate of DA cells and preserving DA termini, which conferred protection on DA neurons (Tönges et al., 2012). 6-OHDA (dopaminergic denervation) mice models often manifest with involuntary motor disorders owing to the increased expression of RhoA/ROCK mRNA in the substantia nigra and striatum, and treatment with high concentrations of fasudil was found to elevate the level of DOPAC (mainly from newly synthesized DA; Tatenhorst et al., 2014). This indicates that high concentrations of fasudil could potentially enhance the regeneration of DA neurons. While L-DOPA, a precursor used in dopamine replacement therapy, remains the main treatment for PD, it can cause movement disorders. In L-DOPA-induced PD rat models, fasudil treatment alleviated this movement disorder by inhibiting the RhoA/ROCK pathway and increasing the production of proinflammatory factors (Lopez-Lopez et al., 2020). This effect could result from microglia polarizing toward MPP-damaged dopaminergic neurons. However, timely administration of fasudil can inhibit its polarization, shifting proinflammatory M1 microglia to an anti-inflammatory M2 phenotype, thereby safeguarding neurons from phagocytosis damage (Barcia et al., 2012; Zhao et al., 2015). In conclusion, fasudil can inhibit α-Syn via multiple mechanisms and targets and improves neurite growth and DA neurons to prevent inflammation in PD.

### 5.3 Huntington’s disease

The R6/2 mice model is a commonly used HD model that exhibits symptoms of progressive retinopathy. Vitreous injection of fasudil directly targeted retinal neurons to inhibit the Htt binding to ROCKs, which in turn prevented the phosphorylation of Profilin and attenuated protein aggregation and neurotoxicity (Charvin et al., 2005). Rats with 3-nitropropionic acid (3-NP)-induced HD exhibit severe mitochondrial dysfunction and striatal degeneration. Fasudil treatment mitigated the pathological effects of 3-NP, reducing mitochondrial dysfunction and neuroinflammation, and the expression of oxidative stress and inflammation, ultimately improving HD symptoms (Ahmed et al., 2016). Although the available evidence suggests that fasudil may be an effective treatment for HD, its application in HD has not been sufficiently clarified, necessitating further investigations.

### 5.4 Amyotrophic lateral sclerosis

ROCKs have been implicated in neuronal death *in vivo* and *in vitro* settings. Fasudil treatment regulated the PTEN/AKT pathway via blocking the activity of ROCKs, thereby decreasing SOD1^G93A^-induced motor neuron cell death and delaying disease progression (Takata et al., 2013). In addition, in SOD1^G93A^ mice, it was observed that fasudil administration before ALS symptoms improved motor function and prolonged life cycle (Tönges et al., 2014). However, in symptomatic mice, fasudil only improved motor function in male mice (Günther et al., 2017). Therefore, future investigations are needed to clarify the associated mechanisms.

### 5.5 Multiple sclerosis

The commonly used MS models are the experimental autoimmune encephalomyelitis (EAE) mice. Pathological changes that accompany EAE include inflammatory cell infiltration and demyelination. It was reported that fasudil significantly inhibited inflammatory response and transformed inflammatory factors such as IL-17 into anti-inflammatory factors such as IL-10, thereby reducing inflammation (Liu et al., 2024). Myelin destruction leads to the enrichment of microglia and phagocytosis of myelin fragments. Fasudil enhances the TREM2/DAP12 pathway, activating microglia to clear myelin debris and stimulating the production of neurotrophic factors, which in turn supports the formation and maturation of OPCs in demyelinated mice (Ding et al., 2021). These data suggest that fasudil may become an alternative treatment for MS, but more evidence is needed before it can be applied in clinical practice.

## 6 Conclusion

The pathogenesis of NDDs remains a challenging and intricate issue in the medical field. Emerging evidence suggests that dysregulated ROCK activity may contribute to the degeneration of the nervous system. However, considering the high similarity of amino acid sequences between ROCK1 and ROCK2, the various inhibitors of ROCKs developed in many studies may have some limitations. In addition, other studies have demonstrated that fasudil confers neuroprotection and repair and can effectively reduce neuronal damage. However, research from recent studies has shown that the inhibition of ROCKs by fasudil is mainly tested in AD and PD. Therefore, future studies should investigate the effects of the inhibition of ROCKs by fasudil on other NDDs. In addition, several mechanisms and pathways contribute to the occurrence of NDDs, in which ROCKs may play a role. Therefore, the inhibition of ROCKs by fasudil can accurately target the treatment of neurodegenerative diseases with few side effects, which remain to be further studied. In addition, all existing ROCK inhibitors have a common problem with low target specificity. For example, fasudil inhibits other kinases such as PKA, PKG, PKC, and MLCK, in addition to ROCKs (Koch et al., 2018). Thus, it is imperative to a gain deeper understanding of ROCK’s specific role in NDDs and developing safer, more targeted ROCK inhibitors in future studies.

ROCK1 is mainly expressed in liver, lung, and blood, while ROCK2 is predominantly expressed in the brain, heart, and muscle. Aberrant ROCK expression has been linked to the development of neurodegenerative diseases such as AD, PD, HD, ALS, and MS.

ROCK1 and ROCK contain a kinase domain, central coiled-coil domain containing RBD domain, PH domain, and CRD domain. The binding of Rho to RBD or cleavage of the C-terminal induces self-inhibition of ROCKs, causing the activation of ROCKs. RBD: Rho-Binding domain; CRD: cysteine-rich domain; PH: Pleckstrin-homology domain.

The stimulation of Rho activates ROCKs, which in turn causes protrusion and axon damage through the phosphorylation of LIMK, CRMP2, PTEN, MLCP, and MYPT1, which increases the risk of neurodegenerative diseases. Fasudil can inhibit the activation of ROCKs.
